# Association of symptoms of attention deficit-hyperactivity disorder and impulsive-aggression with severity of suicidal behavior in adult attempters

**DOI:** 10.1038/s41598-019-41046-y

**Published:** 2019-03-14

**Authors:** I. Conejero, I. Jaussent, R. Lopez, S. Guillaume, E. Olié, C. Hebbache, R. F. Cohen, J. P. Kahn, M. Leboyer, P. Courtet, J. Lopez-Castroman

**Affiliations:** 1 Department of Psychiatry, Nimes University hospital, University of Montpellier, Montpellier, France; 2 Inserm, Unit 1061 “Neuropsychiatry: Epidemiological and Clinical Research”, Montpellier, France; 3 Department of Neurology, CHU Montpellier, University of Montpellier, Montpellier, France; 4 Department of Emergency Psychiatry & Acute Care, CHU Montpellier, University of Montpellier, Montpellier, France; 5 Department of Psychiatry and Clinical Psychology, Université de Lorraine and Nancy University Medical Center, Nancy, France; 6 Department of Psychiatry and Addictology, Mondor Hospital, University Paris Est Créteil, Inserm U 955, fondation FondaMental, Créteil, France; 7 Department of Psychiatry and Clinical Psychology, Centre Psychothérapique, Nancy, France

## Abstract

Literature emphasizes the relationship between attention deficit-hyperactivity disorder (ADHD) and suicidal behavior (SB). However, the link between ADHD and the severity of SB is yet to be determined. We investigated the association between a probable diagnosis of ADHD and the severity of SB in 539 hospitalized suicide attempters, and determined the role of comorbid psychiatric diagnoses. The severity of SB was defined as the number of suicide attempts, age at first suicide attempt, seriousness and violence of suicide attempts. A diagnosis of probable adult ADHD (probable ADHD) was defined as the presence of both current ADHD symptoms and ADHD symptoms in childhood. We evaluated the combined effect of high impulsive-aggression levels and probable ADHD. Probable ADHD was not associated with early or frequent suicide attempts after adjustment for psychiatric disorders and treatment intake. High levels of impulsive-aggression increased the risk of an early suicide attempt, particularly in patients with ADHD symptoms, and independently of other clinical factors. The association between serious suicide attempts and probable ADHD remained significant after adjustment. Although ADHD is involved in suicidal vulnerability, psychiatric comorbidities and impulsive-aggression appear to largely explain the severity of SB in adult attempters with ADHD symptoms.

## Introduction

Attention Deficit Hyperactivity Disorder (ADHD) is a highly prevalent condition associated with substantial psychosocial and health burden^1,2^. According to World Health Organization (WHO) surveys, its prevalence averages 2.2% among children worldwide and is followed by adult ADHD in 57% of the cases^3^ although prevalence of adult ADHD is likely to be underestimated^4^.

ADHD has profound consequences on daily functioning^5^. Recent studies have shown higher rates of comorbid Axis I and Axis II disorders in adults with ADHD compared to those without^6^. ADHD is also associated with divorce and impaired interpersonal relationships^7^. Together, poor social and psychiatric conditions associated with ADHD may trigger suicidal ideation and suicidal behaviors (SB) in patients^8–10^.

There is substantial evidence linking ADHD with different types of suicidal behaviors, such as suicide attempts and completed suicide, and suicidal ideation in young individuals and adult populations. In adolescents, a Finnish birth cohort study (n = 457) showed an independent association between ADHD diagnoses and suicidal ideation (or deliberate self-harm) within the last two years^11^. A Taiwanese study also found that ADHD adolescents were more likely to make deliberate self-poisonings than those without ADHD after adjusting for potential confounders^12^. Another study showed that adolescent attempters with ADHD were older, more often bullying perpetrators and showed higher depression levels than non-attempters^13^.

Similarly, the severity of ADHD symptoms has been linked with frequent suicidal ideation in adults. This association may be mediated by difficulties in coping with stressful situations^14^. Suicide attempters show higher ADHD rates than non-attempters, with OR ranging from 2.6 to 7.3^15^. A recent adult psychiatric morbidity survey (7403 respondents) pointed out a positive association between severity of adult ADHD symptoms and the presence of lifetime or past-12 months suicide attempts, even when taking into account psychiatric comorbid conditions^16^. Common genetic factors, mainly related to impulsivity, were suggested to explain the independent association between SB and ADHD^17^. Indeed, results from a previous prospective follow-up study outlined the existence of shared impulsivity traits between patients with ADHD and those attempting suicide^18^.

In summary, some large epidemiological studies have suggested an association between ADHD and SB but their results were not consistently replicated in clinical samples. The severity of SB associated to a probable diagnosis of ADHD in adults as well as the role of comorbid diagnoses is yet to be determined. We hypothesized that the severity of suicide attempts is positively correlated with probable adult ADHD.

Although birth cohort studies have suggested the possibility of late-onset ADHD emerging in adulthood^19,20^, recent findings showed that a large majority of adults meeting criteria for ADHD had other mental disorders and/ or substance use disorder better explaining the symptoms^21^. Hence, we used the term “probable ADHD” for patients with a diagnosis of ADHD in adulthood when patients reported both current ADHD symptoms and ADHD symptoms in childhood.

We studied the characteristics and severity features of suicidal behavior (number of suicide attempts, age of first suicide attempt, seriousness and violence of suicide attempts), as well as the role of potential confounders such as impulsive-aggression traits, in a large sample of adult suicide attempters with and without ADHD symptoms.

## Results

### Sample description

Of the 684 patients with an evaluation of ADHD symptoms, 78 had missing data for characteristics of suicide, 27 for assessment of impulsive-aggression and 40 for adjustment covariables; they were thus excluded from the present study (n = 145). These patients had a higher educational level, were more frequently smokers, and had less lifetime episodes of major depression than the patients included (p < 0.05 for all these comparisons).

The final sample consisted in 539 patients (66.4% females), the median age was 42.86 years (ranging from 18.01 to 83.45). Almost half of them (46.6%; n = 251) lived with a partner at the time of inclusion. The most frequent psychiatric diagnoses were anxiety disorders (73.3%; n = 395), followed by major depressive disorder (65.4%; n = 353), alcohol use disorder (31.9%; n = 172) and bipolar disorder (27.6%; n = 149). Most patients (n = 446, 82.74%) had at least two comorbid diagnoses among bipolar, major depressive, anxiety, substance use, alcohol use or eating disorder. There were 223 early attempters (41.37%) and 181 frequent suicide attempters (33.58%). Violent and serious suicide attempts occurred respectively in 21.34% (n = 115) and 27.27% (n = 147) of the sample. A family history of suicidal behaviors was reported by 248 (46.01%) patients.

Childhood ADHD symptoms (i.e. positive WURS-25 screening) were found in 161 patients (29.87%). At the time of the inclusion, 254 patients (47.12%) presented with current ADHD symptoms, defined as a positive ASRS screening. Ninety (16.70%) patients screened positive for both childhood and current ADHD symptoms and were thus classified as probable ADHD diagnoses.

Adults with high impulsive-aggression level (BDHI scores >47) were more likely to be screened positive for current ADHD symptoms (59.4%; p = 0.0001).

### Age at first suicide attempt

Compared to late attempters, early attempters were significantly more likely females, and less frequently in couple. They were younger at the time of evaluation and more frequently tobacco users (p = 0.005 for both comparisons). Early attempters were also more frequently diagnosed with anxiety disorder (p = 0.008), substance use and eating disorders (p < 0.0001 for both comparisons) and were prescribed mood stabilizers more often. Finally, high levels of impulsive-aggression were more frequent in early attempters (p < 0.0001) (Supplementary Table S1).

Subsequent analyses were thus adjusted for these factors except for age at inclusion to avoid over-adjustment with age at first suicide attempt (Table 1). The effect of impulsive-aggression was studied independently.

**Table 1:** Age at first suicide attempt according to ADHD screens, in childhood (WURS) and adulthood (ASRS), and impulsive-aggression levels.

Variable	Age at 1^st^ suicide attempt									
	>26 N = 316		≤26 N = 223		Model 0		Model 1		Model 2	
	n	%	n	%	OR [95% CI]	P-value	OR [95% CI]	P-value	OR [95% CI]	P-value
Current ADHD	130	41.14	124	55.61	1.79 [1.27; 2.53]	0.001	1.67 [1.16; 2.40]	0.006	1.57 [1.06; 2.33]	0.03
**ADHD screen**										
No ADHD	237	75.00	141	63.23	1	0.01	1	0.02	1	0.50
Probable ADHD	42	13.29	48	21.52	1.92 [1.21; 3.05]		1.81 [1.12; 2.92]		1.36 [0.81; 2.29]	
Impulsive-aggression	77	24.37	93	41.70	2.22 [1.53; 3.21]	<0.0001	2.21 [1.50; 3.24]	<0.0001	1.96 [1.28; 2.98]	0.002
**Probable ADHD/ Level of impulsive aggression**										
No/Low	155	49.05	61	27.35	1	<0.0001	1	<0.0001	1	0.003
No/High	31	9.81	38	17.04	3.11 [1.78; 5.45]		2.85 [1.61; 5.07]		2.40 [1.27; 4.52]	
Yes/Low	84	26.58	69	30.94	2.09 [1.35; 3.22]		1.85 [1.17; 2.93]		1.70 [1.04; 2.79]	
Yes/High	46	14.56	55	24.66	3.04 [1.86; 4.96]		2.92 [1.75; 4.86]		2.55 [1.46; 4.44]	

Model 0: Crude association.

Model 1: Model adjusted for study site, gender, living in couple.

Model 2: Model adjusted for all covariates in model 1 plus smoking status, anxiety disorder, eating disorder, substance use disorder and treatment intake: Benzodiazepine, antidepressant, mood Stabilizer and antipsychotic.

Current ADHD: Positive ASRS screen (≥4).

Probable ADHD: Positive WURS screen (>46) and ASRS screen (≥4).

Both current ADHD symptoms and probable ADHD were associated with a young age at first suicide attempt after adjustment for socio-demographic variables and study site (model 0 and model 1). The association between current ADHD symptoms and being early attempter remained significant after adjustment for smoking status, anxiety, eating, substance use disorders and treatment intake (benzodiazepine, antidepressant, mood stabilizer and antipsychotic) (model 2 OR 1.57, 95%CI [1.06–2.33]). In contrast, the association between a young age at first suicide attempt and probable ADHD disappeared when considering psychiatric disorders (anxiety and eating disorder), substance use disorders and treatment intake (model 2 OR 1.36, 95%CI [0.81–2.29]).

High levels of impulsive-aggression were associated with early suicide attempts in unadjusted and adjusted models (model 2 OR 1.96, 95%CI [1.28–2.98]). The strength of the association between impulsive-aggression and early suicide attempts increased in patients with probable ADHD (model 2 OR 2.55, 95%CI [1.46–4.44]).

### Number of suicide attempts

Compared to non-frequent suicide attempters, frequent attempters were associated with female gender (p = 0.0002), eating disorder (p < 0.0001), bipolar disorder (p = 0.0001) and a positive family history of SB (p = 0.03). They also displayed higher impulsive-aggression scores (p = 0.02) and were less likely to be diagnosed with major depressive disorder (p = 0.006) or treated with antidepressants (p = 0.02). However, they were more often treated with mood stabilizers than non-frequent suicide attempters (p = 0.0003) (Supplementary Table S1).

Probable ADHD was associated with frequent suicide attempts. These associations remained significant after adjustment for socio-demographic variables and study site (model 0 and model 1) but disappeared when controlling for psychiatric comorbidities (bipolar disorder, major depressive episode, eating disorder), familial history of SA, alcohol use disorder, antidepressant, mood stabilizers and antipsychotic treatment, (model 2 OR 1.62, 95%CI [0.96–2.73]) (Table 2). High impulsive-aggression levels were not associated with frequent suicide attempts.

**Table 2:** Number of suicide attempts (SA) according to ADHD screens, in childhood (WURS) and adulthood (ASRS), and impulsive-aggression levels.

Variable	Number of SA									
	1–2 N = 358		≥3 N = 181		Model 0		Model 1		Model 2	
	n	%	n	%	OR [95% CI]	P-value	OR [95% CI]	P-value	OR [95% CI]	P- value
Current ADHD	163	45.53	91	50.28	1.21 [0.85;1.73]	0.30	1.11 [0.77;1.61]	0.57	0.98 [0.65;1.47]	0.92
**ADHD screen**										
No ADHD	265	74.02	113	62.43	1	0.02	1	0.03	1	0.17
Probable ADHD	51	14.25	39	21.55	1.79 [1.12;2.87]		1.85 [1.14;3.00]		1.62 [0.96;2.73]	
Impulsive-aggression	102	28.49	68	37.57	1.51 [1.03;2.20]	0.03	1.60 [1.08;2.37]	0.02	1.40 [0.91;2.15]	0.12
**Probable ADHD/Level of impulsive aggression**										
No/Low	158	44.13	58	32.04	1	0.02	1	0.05	1	0.28
No/High	37	10.34	32	17.68	2.36 [1.34;4.13]		2.43 [1.36;4.33]		1.86 [0.98;3.52]	
Yes/Low	98	27.37	55	30.39	1.53 [0.98;2.39]		1.37 [0.86;2.18]		1.11 [0.67;1.84]	
Yes/High	65	18.16	36	19.89	1.51 [0.91;2.50]		1.52 [0.90;2.56]		1.27 [0.72;2.23]	

Model 0: Crude association.

Model 1: Model adjusted for study site, gender and age.

Model 2: Model adjusted for all covariates in model 1 plus bipolar disorder, depression, eating disorder, alcohol use disorder, familial history of SA and treatment intake: ATD, Mood Stabilizer and Antipsychotic.

Current ADHD: Positive ASRS screen (≥4).

Probable ADHD: Positive WURS screen (>46) and ASRS screen (≥4).

### Violent suicide attempts

Violent suicide attempters were less often females (<0.0001), had lower educational level (p = 0.01) and were more likely to receive an anxiety disorder diagnosis (p = 0.002) or antidepressant treatment (p = 0.007) than non-violent attempters. However, they received antipsychotics more often (p = 0.008) (Supplementary Table S2). No significant association was found with impulsive-aggression or current ADHD symptoms (Table 3).

**Table 3:** Violent suicide attempt according to ADHD screens, in childhood (WURS) and adulthood (ASRS), and impulsive-aggression levels.

Variable	Violent suicide attempts									
	No N = 424		Yes N = 115		Model 0		Model 1		Model 2	
	n	%	n	%	OR [95% CI]	P-value	OR [95% CI]	P-value	OR [95% CI]	P- value
Current ADHD	205	48.35	49	42.61	0.79 [0.52;1.20]	0.27	1.00 [0.64;1.56]	0.99	1.06 [0.66;1.69]	0.80
**ADHD screen**										
No ADHD	296	69.81	82	71.30	1	0.80	1	0.60	1	0.72
Probable ADHD	70	16.51	20	17.39	1.03 [0.59;1.79]		1.07 [0.59;1.93]		1.01 [0.54;1.89]	
Impulsive-aggression	139	32.78	31	26.96	0.76 [0.48;1.20]	0.23	0.73 [0.45;1.19]	0.21	0.71 [0.42;1.19]	0.19
**Probable ADHD/level of impulsive aggression**										
No/Low	162	38.21	54	46.96	1	0.39	1	0.65	1	0.57
No/High	57	13.44	12	10.43	0.63 [0.32;1.26]		0.70 [0.33;1.45]		0.74 [0.34;1.62]	
Yes/Low	123	29.01	30	26.09	0.73 [0.44;1.21]		1.04 [0.61;1.78]		1.18 [0.66;2.09]	
Yes/High	82	19.34	19	16.52	0.70 [0.39;1.25]		0.77 [0.41;1.45]		0.77 [0.39;1.49]	

Model 0: Crude association.

Model 1: Model adjusted for study site, gender, age, educational level.

Model 2: Model adjusted for all covariates in model 1 plus anxiety disorder, Eating disorder and treatment intake: ATD, Antipsychotic.

Current ADHD: Positive ASRS screen (≥4).

Probable ADHD: Positive WURS screen (>46) and ASRS screen (≥4).

### Serious suicide attempts

Serious suicide attempters were associated with older age (p = 0.02), eating disorder (p = 0.02), and antipsychotic treatment (p = 0.005) compared to patients who did not make a serious attempt (Supplementary Table S2).

Serious suicide attempters were more likely to have a probable ADHD (model 2: OR 1.96 95%CI [1.17–3.29]). No association was found with impulsive-aggression (Table 4).

**Table 4:** Serious suicide attempt according to ADHD screens, in childhood (WURS) and adulthood (ASRS), and impulsive-aggression levels.

Variable	Serious suicide attempts									
	No N = 392		Yes N = 147		Model 0		Model 1		Model 2	
	n	%	n	%	OR [95% CI]	P-value	OR [95% CI]	P-value	OR [95% CI]	P- value
Current ADHD	179	45.66	75	51.02	1.24 [0.85;1.81]	0.27	1.29 [0.87;1.92]	0.20	1.28 [0.85;1.94]	0.24
**ADHD screen**										
No ADHD	286	72.96	92	62.59	1	0.02	1	0.01	1	0.03
Probable ADHD	55	14.03	35	23.81	1.98 [1.22;3.21]		2.09 [1.27;3.45]		1.96 [1.17;3.29]	
Impulsive-aggression	118	30.10	52	35.37	1.27 [0.85;1.90]	0.24	1.40 [0.92;2.12]	0.11	1.35 [0.88;2.07]	0.18
**Probable ADHD/Level of impulsive aggression**										
No/Low	166	42.35	50	34.01	1	0.36	1	0.08	1	0.28
No/High	47	11.99	22	14.97	1.55 [0.86;2.82]		1.73 [0.93;3.22]		1.69 [0.88;3.23]	
Yes/Low	108	27.55	45	30.61	1.38 [0.86;2.21]		1.44 [0.88;2.35]		1.45 [0.86;2.42]	
Yes/High	71	18.11	30	20.41	1.40 [0.82;2.39]		1.58 [0.91;2.75]		1.53 [0.87;2.70]	

Model 0: Crude association.

Model 1: Model adjusted for study site, gender, age.

Model 2: Model adjusted for all covariates in model 1 plus Eating disorder and treatment intake: Mood Stabilizer Antipsychotic.

Current ADHD: Positive ASRS screen (≥4).

Probable ADHD: Positive WURS screen (>46) and ASRS screen (≥4).

## Discussion

In this study, we examined various aspects of SB severity and their relationship with lifetime ADHD symptoms and impulsive-aggression levels in a large and well-characterized sample of adult inpatients with a history of suicide attempts. While the associations of young age at first suicide attempt and frequent suicide attempts with probable adult ADHD disappeared when psychiatric disorders and treatment prescriptions were considered, the association between serious suicide attempts and probable ADHD remained significant. Probable ADHD did not impact the violence of lifetime suicide attempts.

High levels of impulsive-aggression were associated with early suicide attempts, independently of the psychiatric diagnoses, current treatment and ADHD status. The combined effect of impulsive-aggression and probable ADHD increased the risk of making an early suicide attempt. While comorbid psychiatric diagnoses moderated the association between early onset of SB and positive screens for ADHD, high impulsive-aggression levels appeared to be by and large the most important determinant of an early onset of SB in our sample.

Impulsivity is a diagnostic characteristic of ADHD^22^ and impulsive-aggression is a frequent comorbidity in ADHD children^23^ that also characterizes many suicide attempters. Impulsivity, hostility and aggression constructs are also highly correlated and share common features, especially among suicide attempters^24^. Thus, we considered the impulsive-aggression dimension as an independent variable. Our findings fit other results reporting the role of impulsive traits in SB among ADHD populations. Girls diagnosed with a combined type of ADHD (i.e. with attention deficit and hyperactivity) were at higher risk to attempt suicide and self-harm than those with an inattentive type^25^. Children with the hyperactive-impulsive ADHD subtype were at greater risk of attempting suicide than healthy controls, contrary to children with the inattentive subtype^18^.

Previous epidemiological studies have suggested the existence of an independent link between ADHD and the risk of SB, i.e. after adjustment for associated psychiatric disorders. A Swedish cohort including over 50 000 subjects with ADHD showed that they were more likely to attempt (OR = 3.6) and complete suicide (OR = 5.9) than matched controls^17^. Along the same line, the study led by Stickley *et al*.^16^ in a representative sample of the general population (n = 7403) found that the odds for a lifetime suicide attempt ranged from 1.6 to 2.4 in subjects with ADHD symptoms compared to those without^16^. Accordingly, our results show that probable ADHD is associated with highly lethal suicide attempts (leading to admission in an intensive care unit) independently of comorbid psychiatric disorders, treatment or impulsive-aggression levels. Serious suicide attempters, if they survive, often complete suicide later in life^26,27^.

The importance of comorbid substance use disorder in the severity features of SB are consistent with results of previous studies. Kelly *et al*.^28^, reported increased odds ratios for lifetime suicide attempts among male adolescents with comorbid ADHD and substance use disorder compared with those with substance use disorder only (OR = 2.8; CI = 1.2–6.2)^28^. Substance use may exacerbate ADHD symptoms leading to violent acts such as suicide attempts^29^, and may contribute to a poorer prognosis of ADHD^30^. A cross sectional self-report survey on 4 938 Turkish students showed that lifetime drug and alcohol use was associated with more ADHD symptoms^31^. ADHD may lead to an earlier onset and longer duration of substance use^32,33^, which in turn increase the risk for suicidal ideation and SB among adolescents^34^. Indeed, early initiation of substance use is associated with risk factors for suicide in high school students, including suicidal ideation and suicide attempts^35^. The prospective Minnesota twin family study showed that the hyperactivity/impulsivity component of ADHD, but not inattention, predicted the onset of substance use by 18^36^. Hence, higher impulsivity traits in patients with co-existing ADHD and substance use may facilitate the early onset of SB.

Similarly, the association between ADHD status and frequent or early suicide attempts was moderated by mood disorders in our study. Indeed, comorbidity between bipolar disorder and ADHD is frequent^37,38^ and literature shows greater incidence of suicide attempts in patients with ADHD and bipolar disorder than in patients with only bipolar disorder (3% vs 1.1%, p = 0.005)^39^. Depression mediates also the relationship between symptom severity of ADHD and suicidal ideation and SB in adults^14^.

Finally, current psychotropic treatment intake also mediates the relationship between ADHD status and frequent or early suicide attempts. An increased risk for suicidal ideation has been reported in the meta-analysis conducted by Bangs *et al*.^40^ in pediatric patients treated with atomoxetine, but recent cohort studies and meta-analysis did not find any significant relationship between atomoxetine or methylphenidate use and the emergence of suicide attempts or suicide deaths during follow-up^41–44^. Furthermore, participants treated with psychotropic non-stimulant medication were more likely to have a past history of suicidal behaviors, with earlier and more frequent suicide attempts than those admitted without psychotropic treatment. Indeed, ADHD worsens the severity and the outcome of comorbid psychiatric diagnoses^45^. Individuals with ADHD show earlier onset of bipolar disorder and major depressive mood disorder compared with those without ADHD^38^. Additionally, patients with ADHD comorbid with bipolar disorder were more frequently depressed and had shorter periods of wellness^46^. Hence, patients with bipolar disorder or depression comorbid to ADHD may demonstrate poorer outcome resulting in more frequent and earlier suicidal behaviors, and may be more likely to need psychotropic treatment at the time of evaluation.

Our findings should be interpreted in light of some methodological issues: (i) our sample is composed of patients recruited in the aftermath of a suicide attempt and treated in a post-acute care unit; ii) we did not perform a structured clinical interview to confirm the diagnosis of ADHD in patients with positive screening; (iii) we did not perform objective neuropsychological testing to assess executive functioning, memory and motivation impairments, these factors potentially mediating the relationships between ADHD, impulsive-aggression symptoms and characteristics of suicidal behaviors; iv) the retrospective assessment of ADHD symptoms in childhood with the WURS may introduce a recall bias; and iv) the independent use of ASRS leads to a high rate of false positive cases, 47.1% in our study, especially in patients with bipolar disorder or substance use disorders. However, the use of WURS scale has the advantage of evaluating ADHD symptoms before the onset of most psychiatric disorders, and we used a conservative cut-off (>46) which is adapted to psychiatric populations. The combination of both ASRS and WURS found a rate of probable ADHD diagnosis (16.7%) similar to those obtained in other clinical samples of patients with psychiatric diseases^47^.

This is the first study to evaluate the association between probable ADHD and SB severity in a large and well-characterized clinical sample of suicide attempters. ADHD was evaluated through two validated specific scales, a method which has been previously applied to detect adult patients with ADHD^48^. Importantly, results were adjusted by psychopharmacological treatments and psychiatric comorbidities, including substance use disorders.

In summary, our results improve the characterization of SBs among ADHD patients. The association between ADHD and severity features of suicide attempts appears to be driven to a large extent by psychiatric comorbidities and impulsive-aggression levels, especially concerning early suicide attempts. Overall, our results suggest that impulsive-aggression levels predict severity of SB in ADHD and should be systematically controlled in suicidal patients with ADHD. However, ADHD symptoms persisting into adulthood may predict suicide attempts of high medical lethality independently of other clinical factors. These points deserve further research in prospective samples.

## Methods

### Population

Patients included in the present study were recruited as part of a multi-site study involving three French cities: Nancy, Creteil, and Montpellier. Overall, 684 consecutive French speaking adult patients admitted in post-acute care units after a suicide attempt were recruited between 2005 and 2016 and received evaluation of ADHD and impulsive-aggression symptoms during the first week of their hospitalization. Patients were included after receiving a full explanation of the nature of the study and providing their written informed consent. All experimental methods were carried out in accordance with the ethical guidelines determined by the National Ministry of Health, Labour and Welfare and the Declaration of Helsinki. This study was approved by the local ethics committee (CPP Sud Mediterranée IV, CHU Montpellier, France). Patients with current psychotic symptoms or lifetime diagnosis of schizophrenia were not included in the present study. All patients had a structured interview by trained psychiatrists or psychologists.

### Evaluation

#### ADHD symptoms assessment

Symptoms of ADHD were assessed with two validated self-report questionnaires. The *Wender Utah Rating Scale (WURS)*, a 61-item retrospective self-report questionnaire of childhood symptoms. Twenty-five items assess the presence of ADHD symptomatology in childhood^49^. Each item is measured on a five-point Likert scale (0–4), and item responses are summed. The cut-off score of 46/100, which is adapted to screen ADHD in psychiatric populations^50^, was used^51^.

Patients also completed the screener version of the *Adult ADHD Self-Report Scale (ASRS)*^52^, based entirely on the six items with the most stable psychometric properties of the DSM-IV-TR “A” criteria. The World Health Organization (WHO) recommended ASRS for screening of ADHD symptoms in adults and it has been used in numerous studies for the categorization of ADHD in adult patients^16,53^. According to the ASRS screener, patients with at least four out of six items rated with a pathological intensity were categorized as having current ADHD symptoms.

Patients with both childhood and current ADHD symptoms, according to WURS and ASRS scales respectively, were classified as having a probable adult ADHD diagnosis (hereafter probable ADHD)^48^.

#### Suicidal behavior characteristics

SB was assessed with the Columbia Suicide History Form^54^ and Section O of the Diagnostic Interview for Genetics Studies (DIGS)^55^. A suicide attempt was defined according to the National Institute of Mental Health as a self-destructive behavior with some degree of intent to end one’s own life^56^. We defined four principal outcomes to characterize the severity of lifetime SB: the frequency of suicide attempts, the age at first suicide attempt, the seriousness and the violence of suicide attempts^57,58^.

Age at first suicide attempt was defined as the age at which the patient first made a suicide attempt and categorized according to a previously established cut-off (>26 and ≤26 years)^59^. A frequent suicide attempter was defined as having more than two lifetime suicide attempts.

Suicide attempts were considered violent if one of the following methods were used: hanging, firearms, cutting requiring surgical care, throwing oneself under a train and jumping from heights. Conversely, drug overdose and superficial wrist cutting were considered to be non-violent^58,60^. Suicide attempts were considered as serious if admission to intensive care unit was needed^61^. A family history of SB was also assessed.

#### Socio-demographic, lifestyle and psychological measures

All patients had a structured clinical interview to assess socio-demographic and lifestyle variables, such as age at inclusion, gender, tobacco use, marital status, and educational level. The main psychiatric diagnoses (i.e anxiety disorders, major depressive disorder, bipolar disorder, eating disorders, alcohol use disorder and other substance use disorders) were assessed with the Mini-International Neuropsychiatric Interview (MINI)^62^. At the time of inclusion, 493 (95.73%) patients were taking psychotropic drugs (66.60% antidepressants, 51.84% benzodiazepines, 48.74% antipsychotic agents and 22.52% mood stabilizers).

#### Impulsive-aggression assessment

Traits of impulsive-aggression were assessed using the Buss-Durkee Hostility Inventory (BDHI), a 75 items self-report questionnaire^63^. In the absence of validated pathological thresholds, the BDHI total score was divided into tertiles. A cut-off of 47 (corresponding to the highest tertile of the distribution) indicates a high impulsive-aggression level.

### Statistical analysis

Associations between sociodemographic, lifestyle, comorbid psychiatric disorders, impulsive-aggression levels, and SB severity features (number of suicide attempts, age at first suicide attempt, seriousness and violence of the suicide attempts) were analyzed using Chi-square test (for categorical variables) and Student’s t-test (for continuous variables). Study site and variables associated with SB severity features (at p < 0.15) were included in logistic models to estimate adjusted odds ratios (OR) and their 95% confidence intervals (CI) for childhood and current ADHD symptoms, and probable ADHD. Significance level was set at p < 0.05. Statistical analyses were performed using SAS statistical software (version 9.4; SAS Inc, Cary, NC).

## Supplementary information

Dataset 1

## Data Availability

The datasets generated during and/or analysed during the current study are available from the corresponding author on reasonable request.

## References

[1] Biederman J (2005). Attention-deficit/hyperactivity disorder: a selective overview. Biol. Psychiatry.

[2] Karlsdotter K (2016). Burden of illness and health care resource utilization in adult psychiatric outpatients with attention-deficit/hyperactivity disorder in Europe. Curr. Med. Res. Opin..

[3] Fayyad, J. *et al*. The descriptive epidemiology of DSM-IV Adult ADHD in the World Health Organization World Mental Health Surveys. *ADHD Atten*. *Deficit Hyperact*. *Disord*., 10.1007/s12402-016-0208-3 (2016). 10.1007/s12402-016-0208-3 PMC5325787 27866355

[4] Ginsberg, Y., Quintero, J., Anand, E., Casillas, M. & Upadhyaya, H. P. Underdiagnosis of attention-deficit/hyperactivity disorder in adult patients: a review of the literature. *Prim*. *Care Companion CNS Disord*. **16** (2014). 10.4088/PCC.13r01600 PMC4195639 25317367

[5] Küpper T (2012). The negative impact of attention-deficit/hyperactivity disorder on occupational health in adults and adolescents. Int. Arch. Occup. Environ. Health.

[6] Cumyn L, French L, Hechtman L (2009). Comorbidity in adults with attention-deficit hyperactivity disorder. Can. J. Psychiatry Rev. Can. Psychiatr..

[7] Bouchard G, Saint-Aubin J (2014). Attention deficits and divorce. Can. J. Psychiatry.

[8] Cho S-C (2008). Associations between symptoms of attention deficit hyperactivity disorder, depression, and suicide in Korean female adolescents. Depress. Anxiety.

[9] Biederman J (2008). New insights into the comorbidity between ADHD and major depression in adolescent and young adult females. J. Am. Acad. Child Adolesc. Psychiatry.

[10] James A, Lai FH, Dahl C (2004). Attention deficit hyperactivity disorder and suicide: a review of possible associations. Acta Psychiatr. Scand..

[11] Hurtig T, Taanila A, Moilanen I, Nordström T, Ebeling H (2012). Suicidal and self-harm behaviour associated with adolescent attention deficit hyperactivity disorder-a study in the Northern Finland Birth Cohort 1986. Nord. J. Psychiatry.

[12] Chou I-C, Lin C-C, Sung F-C, Kao C-H (2014). Attention-deficit hyperactivity disorder increases the risk of deliberate self-poisoning: A population-based cohort. Eur. Psychiatry.

[13] Chou W-J, Liu T-L, Hu H-F, Yen C-F (2016). Suicidality and its relationships with individual, family, peer, and psychopathology factors among adolescents with attention-deficit/hyperactivity disorder. Res. Dev. Disabil..

[14] Taylor MR, Boden JM, Rucklidge JJ (2014). The relationship between ADHD symptomatology and self-harm, suicidal ideation, and suicidal behaviours in adults: a pilot study. Atten. Deficit Hyperact. Disord..

[15] Impey M, Heun R (2012). Completed suicide, ideation and attempt in attention deficit hyperactivity disorder: Completed suicide in ADHD, ideation and attempt. Acta Psychiatr. Scand..

[16] Stickley A, Koyanagi A, Ruchkin V, Kamio Y (2016). Attention-deficit/hyperactivity disorder symptoms and suicide ideation and attempts: Findings from the Adult Psychiatric Morbidity Survey 2007. J. Affect. Disord..

[17] Ljung T, Chen Q, Lichtenstein P, Larsson H (2014). Common Etiological Factors of Attention-Deficit/Hyperactivity Disorder and Suicidal Behavior: A Population-Based Study in Sweden. JAMA Psychiatry.

[18] Chronis-Tuscano A (2010). Very early predictors of adolescent depression and suicide attempts in children with attention-deficit/hyperactivity disorder. Arch. Gen. Psychiatry.

[19] Caye A (2016). Attention-Deficit/Hyperactivity Disorder Trajectories From Childhood to Young Adulthood: Evidence From a Birth Cohort Supporting a Late-Onset Syndrome. *JAMA*. Psychiatry.

[20] Agnew-Blais JC (2016). Evaluation of the Persistence, Remission, and Emergence of Attention-Deficit/Hyperactivity Disorder in Young Adulthood. *JAMA*. Psychiatry.

[21] Sibley MH (2017). Late-onset ADHD reconsidered with comprehensive repeated assessments between ages 10 and 25. Am. J. Psychiatry.

[22] *Diagnostic and statistical manual of mental disorders: DSM-5*. (American Psychiatric Association, 2013).

[23] Saylor KE, Amann BH (2016). Impulsive Aggression as a Comorbidity of Attention-Deficit/Hyperactivity Disorder in Children and Adolescents. J. Child Adolesc. Psychopharmacol..

[24] Gvion Y, Apter A (2011). Aggression, impulsivity, and suicide behavior: a review of the literature. Arch. Suicide Res. Off. J. Int. Acad. Suicide Res..

[25] Hinshaw SP (2012). Prospective follow-up of girls with attention-deficit/hyperactivity disorder into early adulthood: Continuing impairment includes elevated risk for suicide attempts and self-injury. J. Consult. Clin. Psychol..

[26] Giner L (2014). Violent and serious suicide attempters: one step closer to suicide?. J. Clin. Psychiatry.

[27] Mento C (2016). Serious Suicide Attempts: Evidence on Variables for Manage and Prevent this Phenomenon. Community Ment. Health J..

[28] Kelly TM, Cornelius JR, Clark DB (2004). Psychiatric disorders and attempted suicide among adolescents with substance use disorders. Drug Alcohol Depend..

[29] Yoshimasu, K. S-R and Addictive Disorders as a Risk Factor of Suicide and Homicide among Patients with ADHD: A Mini Review. *Curr*. *Drug Abuse Rev*. (2016). 10.2174/1874473709666160802112215 27492359

[30] Wilens TE, Upadhyaya HP (2007). Impact of substance use disorder on ADHD and its treatment. J. Clin. Psychiatry.

[31] Evren C, Dalbudak E, Evren B, Can Y, Umut G (2014). The severity of attention deficit hyperactivity symptoms and its relationship with lifetime substance use and psychological variables among 10th grade students in Istanbul. Compr. Psychiatry.

[32] Marshal MP, Molina BSG, Pelham WE (2003). Childhood ADHD and Adolescent Substance Use: An Examination of Deviant Peer Group Affiliation as a Risk Factor. Psychol. Addict. Behav. J. Soc. Psychol. Addict. Behav..

[33] Molina BSG, Pelham WE (2014). Attention-Deficit/Hyperactivity Disorder and Risk of Substance Use Disorder: Developmental Considerations, Potential Pathways, and Opportunities for Research. Annu. Rev. Clin. Psychol..

[34] Peltzer K, Pengpid S (2015). Early Substance Use Initiation and Suicide Ideation and Attempts among School-Aged Adolescents in Four Pacific Island Countries in Oceania. Int. J. Environ. Res. Public. Health.

[35] Cho H, Hallfors DD, Iritani BJ (2007). Early Initiation of Substance Use and Subsequent Risk Factors Related to Suicide among Urban High School Students. Addict. Behav..

[36] Elkins IJ, McGue M, Iacono WG (2007). Prospective effects of attention-deficit/hyperactivity disorder, conduct disorder, and sex on adolescent substance use and abuse. Arch. Gen. Psychiatry.

[37] Chen M-H (2013). Higher risk of developing mood disorders among adolescents with comorbidity of attention deficit hyperactivity disorder and disruptive behavior disorder: A nationwide prospective study. J. Psychiatr. Res..

[38] McIntyre, R. S. *et al*. Attention-deficit/hyperactivity disorder in adults with bipolar disorder or major depressive disorder: results from the international mood disorders collaborative project. *Prim*. *Care Companion J*. *Clin*. *Psychiatry* **12** (2010). 10.4088/PCC.09m00861gry PMC2947541 20944770

[39] Lan W-H (2015). Comorbidity of ADHD and suicide attempts among adolescents and young adults with bipolar disorder: A nationwide longitudinal study. J. Affect. Disord..

[40] Bangs ME (2008). Meta-Analysis of Suicide-Related Behavior Events in Patients Treated With Atomoxetine. J. Am. Acad. Child Adolesc. Psychiatry.

[41] Bangs ME, Wietecha LA, Wang S, Buchanan AS, Kelsey DK (2014). Meta-Analysis of Suicide-Related Behavior or Ideation in Child, Adolescent, and Adult Patients Treated with Atomoxetine. J. Child Adolesc. Psychopharmacol..

[42] Linden, S. *et al*. Risk of Suicidal Events With Atomoxetine Compared to Stimulant Treatment: A Cohort Study. *Pediatrics* **137** (2016). 10.1542/peds.2015-3199 PMC4845870 27244795

[43] Liang SH-Y (2018). Suicide risk reduction in youths with attention-deficit/hyperactivity disorder prescribed methylphenidate: A Taiwan nationwide population-based cohort study. Res. Dev. Disabil..

[44] Man KKC (2017). Association of Risk of Suicide Attempts With Methylphenidate Treatment. *JAMA*. Psychiatry.

[45] Katzman, M. A., Bilkey, T. S., Chokka, P. R., Fallu, A. & Klassen, L. J. Adult ADHD and comorbid disorders: clinical implications of a dimensional approach. *BMC Psychiatry* **17** (2017). 10.1186/s12888-017-1463-3 PMC5567978 28830387

[46] Nierenberg AA (2005). Clinical and Diagnostic Implications of Lifetime Attention-Deficit/Hyperactivity Disorder Comorbidity in Adults with Bipolar Disorder: Data from the First 1000 STEP-BD Participants. Biol. Psychiatry.

[47] Kessler RC (2006). The Prevalence and Correlates of Adult ADHD in the United States: Results From the National Comorbidity Survey Replication. Am. J. Psychiatry.

[48] Fond G (2015). Prevalence and smoking behavior characteristics of nonselected smokers with childhood and/or adult self-reported ADHD symptoms in a smoking-cessation program: a cross-sectional study. J. Atten. Disord..

[49] Stein, M. A. *et al*. Psychometric characteristics of the Wender Utah Rating Scale (WURS): reliability and factor structure for men and women. *Psychopharmacol*. *Bull*. (1995). 7491401

[50] Adamis, D. *et al*. Screening for attention deficit–hyperactivity disorder (ADHD) symptomatology in adult mental health clinics. *Ir*. *J*. *Psychol*. *Med*. 1–9, 10.1017/ipm.2017.49 (2017). 10.1017/ipm.2017.49 30124183

[51] Rösler M (2006). Psychopathological rating scales for diagnostic use in adults with attention-deficit/hyperactivity disorder (ADHD). Eur. Arch. Psychiatry Clin. Neurosci..

[52] Murphy KR, Adler LA (2004). Assessing attention-deficit/hyperactivity disorder in adults: focus on rating scales. J. Clin. Psychiatry.

[53] Kessler RC (2005). TheWorld Health Organization Adult ADHD Self-Report Scale (ASRS): a short screening scale for use in the general population. Psychol. Med..

[54] Mann JJ, Waternaux C, Haas GL, Malone KM (1999). Toward a clinical model of suicidal behavior in psychiatric patients. Am. J. Psychiatry.

[55] Preisig M, Fenton BT, Matthey ML, Berney A, Ferrero F (1999). Diagnostic interview for genetic studies (DIGS): inter-rater and test-retest reliability of the French version. Eur. Arch. Psychiatry Clin. Neurosci..

[56] Pearson JL, Stanley B, King CA, Fisher CB (2001). Intervention research with persons at high risk for suicidality: safety and ethical considerations. J. Clin. Psychiatry.

[57] Lopez-Castroman J (2016). Heavy tobacco dependence in suicide attempters making recurrent and medically serious attempts. Drug Alcohol Depend..

[58] Lopez-Castroman J (2011). Distinguishing the relevant features of frequent suicide attempters. J. Psychiatr. Res..

[59] Slama F (2009). Admixture analysis of age at first suicide attempt. J. Psychiatr. Res..

[60] Åsberg M, Träskman L, Thorén P (1976). 5-HIAA in the Cerebrospinal Fluid: A Biochemical Suicide Predictor?. Arch. Gen. Psychiatry.

[61] Guillaume S (2010). Suicide attempt characteristics may orientate toward a bipolar disorder in attempters with recurrent depression. J. Affect. Disord..

[62] Sheehan DV (1998). The Mini-International Neuropsychiatric Interview (M.I.N.I.): the development and validation of a structured diagnostic psychiatric interview for DSM-IV and ICD-10. J. Clin. Psychiatry.

[63] Buss AH, Durkee A (1957). An inventory for assessing different kinds of hostility. J. Consult. Psychol..

