# Supplementary material for: Association of symptoms of attention deficit-hyperactivity disorder and impulsive-aggression with severity of suicidal behavior in adult attempters
Source: Sci Rep. 2019 Mar 14;9:4593. doi: 10.1038/s41598-019-41046-y (PMC6418227; doi:10.1038/s41598-019-41046-y)
Supplement: Supplementary file 1 — Dataset 1 [file 41598_2019_41046_MOESM1_ESM.doc]

**Title:** Association of symptoms of attention deficit-hyperactivity disorder and impulsive-aggression with severity of suicidal behavior in adult attempters.

I Conejero^a*^, I Jaussent^b^, R Lopez^c^, S Guillaume^bd^, C Hebbache^f^, RF Cohen^g^, JP Kahn^eg^, M Leboyer^f^, P Courtet^bd^, J Lopez-Castroman^ab^

Supplementary table S1. Associations between socio-demographic and clinical characteristics with different suicide features: age at the first suicide attempt and number of suicide attempts

|  | Age at the first suicide attempt | | | | |  | | Number of suicide attempts | | | |  | |  |
| --- | --- | --- | --- | --- | --- | --- | --- | --- | --- | --- | --- | --- | --- | --- |
|  | *>26*  *N=316* | | | *≤26*  *N=223* | |  | | *1-2*  *N=358* | | *≥3*  *N=181* | |  | |  |
| Variable | n | | % | n | % | p | | n | % | n | % | P | |  |
| Sex (Female) | 194 | | 61.39 | 164 | 73.54 | 0.003 | | 218 | 60.89 | 140 | 77.35 | 0.0002 | |  |
| Age (years) ^(1)^ | 47.14  [27.18-83.45] | | | 30.99  [18.06-67.43] | | <0.0001 | | 41.91  [18.06-83.45] | | 45.04  [18.42-70.25] | | 0.11 | |  |
| Educational level |  | |  |  |  |  | |  |  |  |  |  | |  |
| ≤9 | 58 | | 18.35 | 40 | 17.94 | 0.70 | | 61 | 17.04 | 37 | 20.44 | 0.28 | |  |
| 10-12 | 115 | | 36.39 | 89 | 39.91 |  | | 131 | 36.59 | 73 | 40.33 |  | |  |
| ≥13 | 143 | | 45.25 | 94 | 42.15 |  | | 166 | 46.37 | 71 | 39.23 |  | |  |
| Living with a partner | 175 | | 55.38 | 76 | 34.08 | <0.0001 | | 171 | 47.77 | 80 | 44.20 | 0.43 | |  |
| Smoking status |  | |  |  |  |  | |  |  |  |  |  | |  |
| Never smoker | 118 | | 37.34 | 73 | 32.74 | 0.005 | | 133 | 37.15 | 58 | 32.04 | 0.28 | |  |
| Current smoker | 130 | | 41.14 | 121 | 54.26 |  | | 158 | 44.13 | 93 | 51.38 |  | |  |
| Past smoker | 68 | | 21.52 | 29 | 13.00 |  | | 67 | 18.72 | 30 | 16.57 |  | |  |
| Bipolar disorder | 82 | | 25.95 | 67 | 30.04 | 0.30 | | 80 | 22.35 | 69 | 38.12 | 0.0001 | |  |
| Major depression | 214 | | 67.72 | 139 | 62.33 | 0.20 | | 249 | 69.55 | 104 | 57.46 | 0.006 | |  |
| Anxiety disorder | 218 | | 68.99 | 177 | 79.37 | 0.008 | | 257 | 71.79 | 138 | 76.24 | 0.27 | |  |
| Eating disorder | 36 | | 11.39 | 59 | 26.46 | <0.0001 | | 43 | 12.01 | 52 | 28.73 | <0.0001 | |  |
| Substance use disorder | 34 | | 10.76 | 65 | 29.15 | <0.0001 | | 62 | 17.32 | 37 | 20.44 | 0.38 | |  |
| Alcohol use disorder | 96 | | 30.38 | 76 | 34.08 | 0.36 | | 106 | 29.61 | 66 | 36.46 | 0.11 | |  |
| Family history of suicidal behavior | 140 | | 44.30 | 108 | 48.43 | 0.34 | | 153 | 42.74 | 95 | 52.49 | 0.03 | |  |
| Impulsive-aggression (BDHI total score) |  | |  |  |  |  | |  |  |  |  |  | |  |
| ≤38 | 136 | | 43.04 | 64 | 28.70 | <0.0001 | | 148 | 41.34 | 52 | 28.73 | 0.02 | |  |
| ]38-47] | 103 | | 32.59 | 66 | 29.60 |  | | 108 | 30.17 | 61 | 33.70 |  | |  |
| >47 | 77 | | 24.37 | 93 | 41.70 |  | | 102 | 28.49 | 68 | 37.57 |  | |  |
| Medication intake at the inclusion | |  | | | | |  | | | | | |  | |
| Benzodiazepines | 166 | | 54.79 | 101 | 47.64 | 0.11 | | 180 | 51.87 | 87 | 51.79 | 0.99 | |  |
| Antidepressants | 212 | | 69.97 | 131 | 61.79 | 0.05 | | 243 | 70.03 | 100 | 59.52 | 0.02 | |  |
| Mood stabilizers | 50 | | 16.50 | 66 | 31.13 | 0.0001 | | 62 | 17.87 | 54 | 32.14 | 0.0003 | |  |
| Antipsychotics | 137 | | 45.21 | 114 | 53.77 | 0.06 | | 159 | 45.82 | 92 | 54.76 | 0.06 | |  |

1. continuous variables were expressed as *median value [minimum value-maximum value)*

Supplementary table S2. Associations between socio-demographic and clinical characteristics and different suicide features: violent suicide attempt and serious suicide attempt.

|  | Violent suicide attempt | | | |  | Serious suicide attempt | | | |  |
| --- | --- | --- | --- | --- | --- | --- | --- | --- | --- | --- |
|  | *No*  *N=424* | | *Yes*  *N=115* | |  | *No*  *N=392* | | *Yes*  *N=147* | |  |
| Variable | n | % | n | % | p | n | % | n | % | P |
| Sex (Female) | 308 | 72.64 | 50 | 43.48 | <0.0001 | 253 | 64.54 | 105 | 71.43 | 0.13 |
| Age (years) ^(1)^ | 42.50 [18.06-83.45] | | 45.35 [18.42-67.43] | | 0.13 | 41.82 [18.06-83.45] | | 45.69 [19.56-70.25] | | 0.02 |
| Educational level |  |  |  |  |  |  |  |  |  |  |
| ≤9 | 75 | 17.69 | 23 | 20.00 | 0.01 | 76 | 19.39 | 22 | 14.97 | 0.29 |
| 10-12 | 149 | 35.14 | 55 | 47.83 |  | 151 | 38.52 | 53 | 36.05 |  |
| ≥13 | 200 | 47.17 | 37 | 32.17 |  | 165 | 42.09 | 72 | 48.98 |  |
| Living as a couple | 202 | 47.64 | 49 | 42.61 | 0.34 | 182 | 46.43 | 69 | 46.94 | 0.92 |
| Smoking status |  |  |  |  |  |  |  |  |  |  |
| Never smoker | 149 | 35.14 | 42 | 36.52 | 0.55 | 145 | 36.99 | 46 | 31.29 | 0.47 |
| Current smoker | 202 | 47.64 | 49 | 42.61 |  | 178 | 45.41 | 73 | 49.66 |  |
| Past smoker | 73 | 17.22 | 24 | 20.87 |  | 69 | 17.60 | 28 | 19.05 |  |
| Bipolar disorder | 116 | 27.36 | 33 | 28.70 | 0.78 | 103 | 26.28 | 46 | 31.29 | 0.25 |
| Major depression | 284 | 66.98 | 69 | 60.00 | 0.16 | 259 | 66.07 | 94 | 63.95 | 0.64 |
| Anxiety disorder | 324 | 76.42 | 71 | 61.74 | 0.002 | 283 | 72.19 | 112 | 76.19 | 0.35 |
| Eating disorder | 82 | 19.34 | 13 | 11.30 | 0.05 | 60 | 15.31 | 35 | 23.81 | 0.02 |
| Substance use disorder | 81 | 19.10 | 18 | 15.65 | 0.40 | 69 | 17.60 | 30 | 20.41 | 0.45 |
| Alcohol use disorder | 132 | 31.13 | 40 | 34.78 | 0.46 | 126 | 32.14 | 46 | 31.29 | 0.85 |
| Family history of suicidal behavior | 192 | 45.28 | 56 | 48.70 | 0.52 | 177 | 45.15 | 71 | 48.30 | 0.51 |
| Impulsive-aggression (BDHI total score) |  |  |  |  |  |  |  |  |  |  |
| ≤38 | 148 | 34.91 | 52 | 45.22 | 0.13 | 145 | 36.99 | 55 | 37.41 | 0.36 |
| ]38-47] | 137 | 32.31 | 32 | 27.83 |  | 129 | 32.91 | 40 | 27.21 |  |
| >47 | 139 | 32.78 | 31 | 26.96 |  | 118 | 30.10 | 52 | 35.37 |  |
| Medication intake at the inclusion |  |  |  |  |  |  |  |  |  |  |
| Benzodiazepines | 214 | 52.58 | 53 | 49.07 | 0.52 | 190 | 51.35 | 77 | 53.10 | 0.72 |
| Antidepressants | 283 | 69.53 | 60 | 55.56 | 0.007 | 243 | 65.68 | 100 | 68.97 | 0.48 |
| Mood stabilizers | 92 | 22.60 | 24 | 22.22 | 0.93 | 75 | 20.27 | 41 | 28.28 | 0.05 |
| Antipsychotics | 186 | 45.70 | 65 | 60.19 | 0.008 | 166 | 44.86 | 85 | 58.62 | 0.005 |

1. continuous variables were expressed as *median value [minimum value-maximum value)*
